# Characterization of Films Based on *Opuntia ficus-indica* and Pectin

**DOI:** 10.1007/s11130-026-01570-5

**Published:** 2026-09-23

**Authors:** Júlia Matos Coqueiro, Júlia Marques Brandão, Stefanny Pereira Atanes, Sibele Santos Fernandes, Deborah Murowaniecki Otero

**Affiliations:** 1 Graduate Program in Food Science, Faculty of Pharmacy, Federal University of Bahia, Campus Ondina, Salvador, Bahia 40170-290 Brazil; 2 Nutrition School, Federal University of Bahia, Campus Canela, Salvador, Bahia 40110907 Brazil; 3 Laboratory of Food Technology, School of Chemistry and Food, Federal University of Rio Grande, Rio Grande, RS Brazil; 4 Graduate Program in Food, Nutrition and Health, Nutrition School, Federal University of Bahia, Campus Canela, Salvador, Bahia 40110907 Brazil

**Keywords:** Polysaccharides, Cactus, Sustainability, Food packaging, Freeze-dried, Byproduct

## Abstract

Plastic packaging is widely used in the food industry, but its persistence in the environment for centuries has driven interest in biodegradable alternatives. This study aimed to develop a biodegradable film using freeze-dried *Opuntia ficus-indica* cladodes in powder form (OFICP) (2 g), glycerol (30%, w/w), and varying amounts of commercial pectin: 0.5 g (F1), 1.0 g (F2), 1.5 g (F3), and 2.0 g (F4). The influence of pectin content on the films’ mechanical, thermal, structural, color, and water-related properties was systematically evaluated, alongside their biodegradability in soil and simulated marine environments. Formulations containing higher amounts of pectin exhibited enhanced mechanical performance, including a 232% increase in tensile strength from F1 (2.62) to F3 (8.69), as well as improved thermal stability. Due to the hydrophilic nature of both pectin and OFICP, the films showed low contact angles (30.29–39.39°) and high moisture content (25.11–35.98%). Considering their hydrophilic nature and water-related properties, the films may be more suitable for applications involving low-moisture food products; however, further studies under different storage and environmental conditions are needed to confirm their suitability. Moreover, under experimental conditions, the films showed progressive visual disintegration in soil, with F1 and F2 exhibiting substantial disintegration by day 12 and all formulations becoming almost completely disintegrated by day 28. Thus, these findings suggest that blending OFICP with commercial pectin produces films with improved properties, with potential applications in food packaging. Furthermore, valorizing *Opuntia ficus-indica* byproducts provides an environmentally responsible approach to agricultural waste management while supporting the development of next-generation bio-based materials for food packaging.

**Supplementary Information:**

The online version contains supplementary material available at https://doi.org/10.1007/s11130-026-01570-5.

## Introduction

The growing concern about the environmental impacts of intensive plastic packaging use has stimulated the search for sustainable alternatives, particularly in the food sector, where material disposal is high [1]. Plastic packaging, mainly derived from non-renewable sources, is non-biodegradable and significantly contributes to environmental pollution and the accumulation of solid waste [2]. In this context, biodegradable films made from natural biopolymers have emerged as promising solutions, combining technological functionality with reduced environmental impact while aligning with the principles of the circular economy and sustainability [3].

Among these materials, plant-derived polysaccharides have attracted particular interest due to their structural diversity and capacity to form continuous polymeric matrices. In this context, *Opuntia ficus-indica*, a cactus species extensively cultivated in semi-arid regions, represents a promising yet underexplored resource. Its cladodes are generated in large quantities during agricultural management and are often discarded or underutilized [4] despite being rich in polysaccharides, dietary fibers, and other functional constituents [5].

Recent studies have demonstrated that materials derived from *Opuntia ficus-indica* cladodes can be used to produce biodegradable films with acceptable mechanical and barrier properties [6, 7]. However, many of these approaches rely on the extraction of mucilage or specific fractions, which increases processing complexity and limits scalability. An alternative strategy is the direct use of whole cladodes after drying, allowing full valorization of the biomass while simplifying processing steps. Freeze-drying enables the preservation of structural components and facilitates the production of homogeneous powders suitable for film formulation.

Despite their potential, bio-based films often require additional molecules in their formulation to optimize characteristics such as flexibility and mechanical resistance. Pectin, a polysaccharide rich in galacturonic acid units and commonly obtained from citrus fruits [5], is an effective option for this purpose. Owing to its intrinsic gelling and film-forming abilities [8, 9], it integrates nicely into biodegradable matrices, enhancing their mechanical, thermal, and barrier performance [10] Thus, its inclusion in film formulations supports the development of materials with improved structural stability and functional efficiency.

Plasticizers are also essential in biopolymer-based films to overcome brittleness and improve flexibility. Glycerol is one of the most employed plasticizers due to its compatibility with hydrophilic polymers and its ability to increase chain mobility [11]. The combination of *Opuntia ficus-indica cladode*-derived material, pectin, and glycerol represents a formulation strategy that leverages the complementary characteristics of these components and may improve film performance.

Although several studies have explored films derived from *Opuntia ficus-indica* or pectin individually, investigations of their combined use remain limited. Understanding how pectin content influences the physicochemical, mechanical, thermal, and barrier properties of films incorporating freeze-dried *Opuntia ficus-indica* cladode powder is essential to advance the development of sustainable packaging materials.

Therefore, this study aimed to develop and characterize biodegradable blend films based on freeze-dried *Opuntia ficus-indica* cladode powder (OFICP), pectin, and glycerol, and to evaluate their physicochemical, mechanical, thermal, and barrier properties in order to assess their suitability for potential food packaging applications.

## Materials and Methods

The materials and methods section are presented as supplementary material.

## Results and Discussion

### Film Appearance

Initially, preliminary film-forming trials were conducted using different concentrations of OFICP and glycerol. However, these formulations resulted in brittle materials that were difficult to detach from the Petri dishes and were not suitable for handling or further characterization. Consequently, a pectin-only formulation (2 g pectin + glycerol) was selected as the control system, serving as a reference matrix for comparison with the OFICP/pectin composite films. Additionally, all composite formulations (F1–F4) produced homogeneous films without visible cracks or air bubbles and were easily handled (Fig. S1).

### Water-related Properties

Water significantly influences both the visual quality and spoilage processes of food, making it a key factor in maintaining safety during storage. To ensure food remains protected, packaging materials must control moisture transfer effectively by acting as barriers against external humidity [15]. Therefore, evaluating water-related properties, such as moisture content, contact angle, water activity (A_w_), and water vapor permeability (WVP), is essential for understanding and optimizing the performance of OFICP/Pectin blended films. Table 1 presents the moisture content, A_w_, WVP, contact angle, and image of a water droplet on the surface of the films.

**Table 1:**
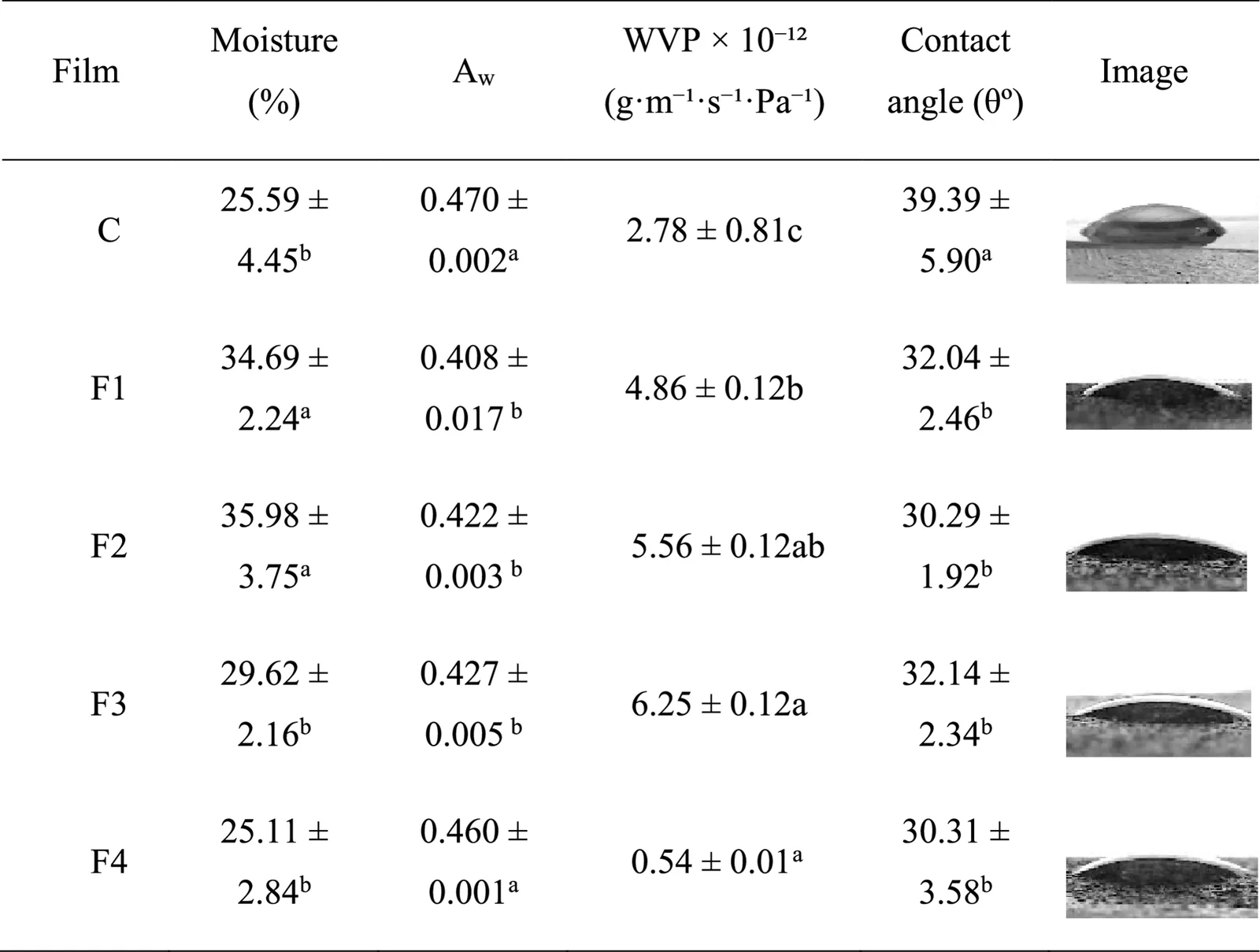
Moisture content, water activity (Aw), water vapor permeability (WVP), contact angle, and image of water droplet on the surface of the films

C = control film (2 g pectin); F1 (0.5 g pectin + 2 g of OFICP), F2 (1.0 g pectin +2 g of OFICP), F3 (1.5 g pectin + 2g of OFICP), F4 (2 g pectin + 2 g of OFICP). Average of three values with standard deviation, the same letter in the column indicates no significant difference between the means by the Tukey test (*p* < 0.05)

The moisture content of the films varied from one formulation to another. Regarding the blend films, the increase in the pectin content up to 1.5 g led to a lower moisture content. This could be explained by the formation of hydrogen-bonding interactions between OFICP and pectin, which reduces the number of –OH that may associate with water [4]. These findings are in line with previous studies. Makhloufi et al. [12] reported that films made from OFICP and 30% (w/w) glycerol had lower moisture content values (20.6%). Similarly, Oudir et al [4] developed films containing OFICP, xanthan gum, and agar, which exhibited a moisture content close to 30%. These results reflect the intrinsic hydrophilicity of both *Opuntia ficus-indica* cladodes and pectin, which limits their ability to resist water penetration and retain structural integrity in humid environments. However, this characteristic also presents a potential advantage for applications where water solubility is desirable, such as in soluble or edible packaging systems.

The incorporation of OFICP influenced A_w_ and WVP values, which followed a trend: as the pectin content increased, water vapor permeability and water activity also increased, indicating a more permeable structure. Water activity ranged from 0.408 in F1 to 0.470 in the control film, which is lower than the values reported by Tristanto et al [16] for pectin-based films (0.614–0.652). Despite the differences, the A_w_ values obtained reflect the low free water content within the film matrix, which is consistent with the physicochemical properties of the polymers used. It should be noted that the differences in film thickness among formulations may have contributed to the observed variations in WVP and mechanical properties. Since the same volume of film-forming solution was cast for all formulations, whereas the solid content varied according to the pectin content, film thickness was not controlled as an independent variable. Therefore, differences in film thickness may also have contributed to the observed variations.

The WVP values ranged from 2.78 (g·m⁻¹·s⁻¹·Pa⁻¹) for the control film, to 0.54 (g·m⁻¹·s⁻¹·Pa⁻¹) for formulation F4. The highest values were observed in formulations F3 and F4, which differed significantly from F1 and the control film. This increase can be attributed to the addition of solids to the formulations, particularly the larger amounts of pectin added to the OFICP itself. Additionally, the hydrophilic character of both the polymers and the plasticizer must be considered, as glycerol’s hygroscopicity promotes stronger interactions with water molecules, enhancing absorption and permeability while diminishing the film’s barrier properties [17].

The contact angle provides insight into the surface wettability of the films [12]. Smaller angles (< 90°) indicate high wettability, whereas larger angles (> 90°) suggest a more hydrophobic surface [13]. All films in this study exhibited contact angles below 90°, confirming their hydrophilic nature. Among them, only the control film showed a significantly higher contact angle (*p* < 0.05), indicating a less wettable surface than the blended formulations. These findings are consistent with previous studies: de Farias et al [18] reported contact angles of 26.94° for starch/OFICP films without pH treatment, while Makhloufi et al [12] observed values of 26.40° for OFICP–agar films containing 30% (w/w) glycerol. Interestingly, de Farias et al. [18] also demonstrated that pH adjustment can strongly influence wettability, reporting a contact angle of 70.85° in starch/OFICP films treated at pH 12. This suggests that pH modification of OFICP could be a promising strategy to tailor surface hydrophobicity in future formulations.

### Opacity and Color of Films

Table S1 presents color parameters and opacity of developed films. The control film presented the highest L* value (90.67), indicating high luminosity (lighter film). Meanwhile, the addition of OFICP significantly reduced L*, especially for F1 (65.83), resulting in darker films. As expected, increasing the pectin content and consequently the total solids further reduced film clarity, producing more opaque and intensely colored films, as visually observed in Fig. S1.

The control film showed a tendency toward red (a* positive), while all films with OFICP showed negative values, indicating a tendency toward green. Regarding the b* color, all films showed a tendency toward yellow (b* positive), which was more pronounced in films containing OFICP. Therefore, the presence of OFICP alters the hue of the red-green axis, shifting it toward green and intensifying the yellow hue of the films.

The total color difference (ΔE*) was lowest in the control film (17.00). With the addition of OFICP, ΔE increased significantly, especially in F1 (54.64), indicating greater contrast than in the control. Compared to the other composite films, control films (C, pure pectin) exhibited higher L* values and lower b* and ΔE values. A higher L* value indicates a brighter film surface [19]. Although film color can directly influence the visual appeal of packaged foods and consumer acceptance [20], the OFICP/Pectin films exhibit a light brownish hue similar to that of kraft paper bags [12], which may not negatively affect their application. Moreover, films with higher opacity could serve as effective barriers against UV-induced lipid oxidation, making them particularly useful for packaging high-fat foods [14].

The total color difference (ΔE*) was lowest in the control film (17.00). With the addition of OFICP, ΔE increased significantly, especially in F1 (54.64), indicating greater contrast than in the control.

Compared to the other composite films, control films (C, pure pectin) exhibited higher L* values and lower b* and ΔE values. A higher L* value indicates a brighter film surface [19]. Although film color can directly influence the visual appeal of packaged foods and consumer acceptance [20], the OFICP/Pectin films exhibit a light brownish hue similar to that of kraft paper bags [12], which may not negatively affect their application. Moreover, films with higher opacity could serve as effective barriers against UV-induced lipid oxidation, making them particularly useful for packaging high-fat foods [14].

The opacity of the films studied varied from 14.94 to 25.07, with the control film having the lowest opacity. All films with OFICP showed high opacity (≈ 22–25%), with no significant difference between F1 and F4. This shows that regardless of the amount of pectin, the incorporation of OFICP considerably increases the opacity of the films. Higher opacity values correspond to a lower degree of transparency. Opacity can be influenced by the size of particles dispersed in the film-forming solution, as larger particles can scatter light more effectively, increasing the turbidity of the film [14].

Overall, the addition of OFICP reduced luminosity and increased yellow (b*) and greenish hues, in addition to increasing opacity. These changes indicate that the natural pigments and constituents of OFICP, combined with pectin, directly impact the color and transparency of the films. In packaging applications, this may be beneficial for protecting light-sensitive foods, but it may limit uses where transparency is desired. Furthermore, Romani et al. [21] consider that color is not a limiting factor in film application; the parameters are more related to appearance and consumer acceptability.

### Film Thickness

**Fig. 1:**
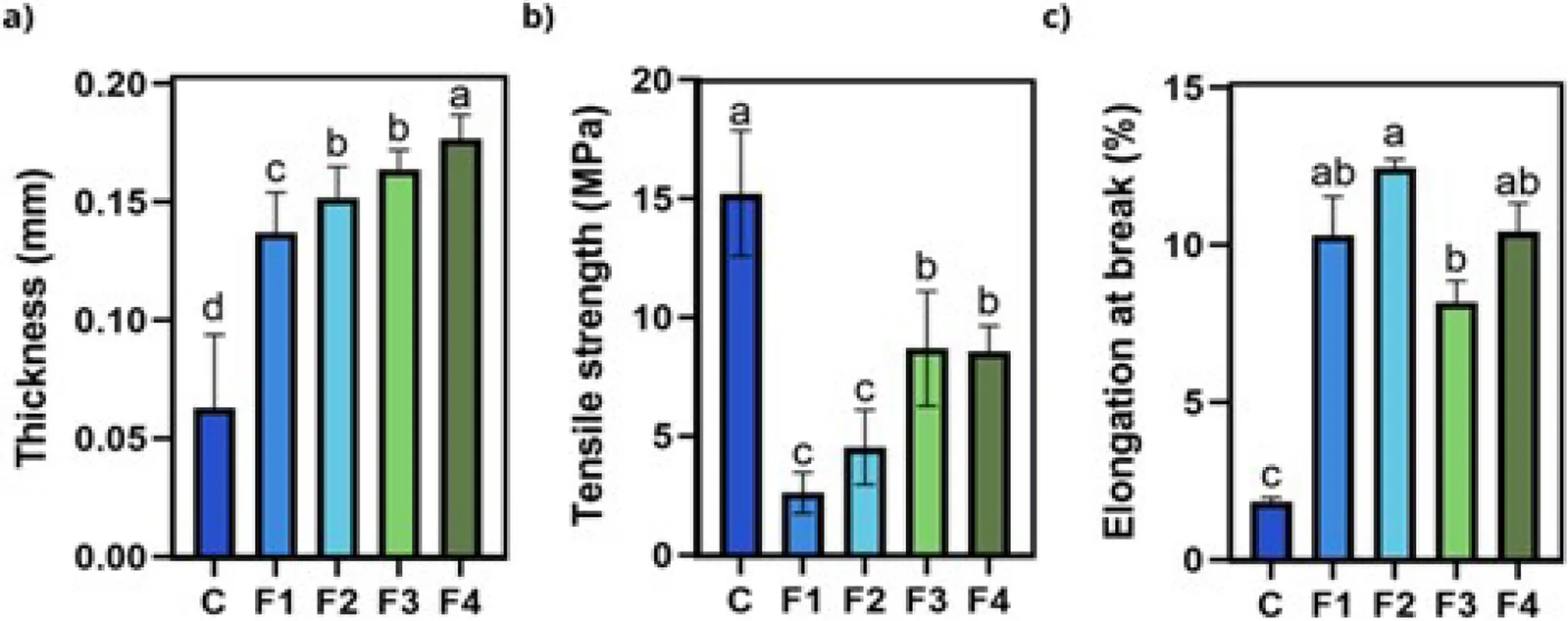
(**a**) Thickness, (**b**) Tensile strength, and (**c**) Elongation at break of OFICP/Pectin-based films

Film thickness plays a crucial role in determining their overall performance, significantly affecting characteristics such as mechanical strength, barrier efficiency, and ease of handling [4]. Figure 1 shows the thickness and mechanical properties of the developed films. As shown in Fig. 1a, the increase in pectin in the blends resulted in thicker films, with thickness values ranging from 0.137 mm for formulation F1 to 0.177 mm for F4. This trend was expected due to the increase in the dry matter content.

### Mechanical Properties

The control formulation, containing 2 g of pectin without OFICP addition, was used as a reference film to evaluate the influence of cladode powder incorporation on the properties of the biodegradable films. Regarding tensile strength (Fig. 1b), the control film (C) exhibited the highest value (15.23 MPa), significantly surpassing all other formulations. In contrast, the tensile strength of the OFICP/Pectin films increased progressively from F1 (2.62 MPa) to F3 (8.69 MPa), with no significant difference between F3 and F4. This plateau suggests that the strengthening effect of pectin may reach a saturation point beyond a certain concentration, after which no further increase in tensile strength is achieved.

*C = control film (2 g pectin); F1= (0.5 g pectin + 2 g of OFICP), F2 = (1.0 g pectin + 2 g of OFICP), F3 = (1.5 g pectin + 2 g of OFICP), F4 = (2 g pectin + 2 g of OFICP).

In comparison to previous studies, the present results demonstrate a notable improvement. De Farias et al. [18] reported a lower tensile strength (3.0 MPa) for films composed of starch and OFICP, while Makhloufi et al. [12] observed a value of 6.7 MPa for films containing agar, OFICP, and 30% glycerol (w/w). These comparisons underscore the potential of moderate pectin concentrations to increase the tensile strength of cactus-based films significantly.

The mechanical properties of the films revealed a typical trade-off between tensile strength and elongation. The control film exhibited the highest tensile strength, indicating that it could withstand greater force before breaking. However, it also showed the lowest elongation at break (1.86%), suggesting a rigid and brittle structure with limited flexibility. In contrast, formulations F1 and F2 demonstrated significantly lower tensile strength but higher elongation values (10.30% and 12.46%, respectively), reflecting a more elastic and ductile nature. As a result, although these films are more susceptible to tearing under strong force, they are better able to stretch and adapt to mechanical stress. These findings underscore the multifunctional role of OFICP in modulating the balance between strength and flexibility in biodegradable film development, with the control film being more resistant, but brittle, and the F2 film being less resistant, but more flexible.

### Thermal Stability

Figure S2 presents the thermal performance of the developed films evaluated using TGA and DSC analyses. Overall, the DSC thermograms of all formulations revealed broad and poorly defined transitions (Fig. S2a), which are characteristic of most amorphous materials. This behavior reflects the high chain mobility and low crystallinity of the polymer matrix of both OFICP and pectin.

No sharp endothermic peaks typically associated with melting points were detected, indicating the absence of a well-defined crystalline phase. Instead, subtle deviations in the baseline of the heat flow curves were observed between approximately 50–100 °C, especially in formulations F3 and F4, which may correspond to the glass transition temperature (Tg) [22, 23]. However, these inflections are not resolved due to the overlapping effect of moisture loss and the intrinsic amorphous nature of the matrix. In this type of system, water can act as a plasticizer, masking the Tg by lowering the temperature at which molecular mobility increases [6]. A slight increase in enthalpy was observed in formulations F3 and F4 compared to the control and F1, suggesting enhanced molecular organization or interactions between the biopolymer chains and pectin. This trend supports the hypothesis of stronger matrix–pectin interactions in films with higher pectin content, which may promote partial rearrangement or more compact structures.

The thermograms for TGA (Fig. S2b) shows three major weight-loss stages: the first one from 50 to 100 °C is characterized by the evaporation of free or physically adsorbed water; the second, observed approximately between 220 and 260 °C, represents the main thermal degradation phase; and the third stage, occurring above 400 °C, is attributed to the breakdown of main chain C-C bonds in the blend [24].

The control sample (C) exhibited a maximum decomposition temperature of 224 °C and reached 50% weight loss at 241 °C, with a total weight loss of 37% at the temperature of maximum mass-loss rate (DTGₘₐₓ) (Fig. S2c). Among the formulations, F1 exhibited a DTGₘₐₓ at 252 °C and reached a 50% weight loss at a slightly higher temperature (254 °C), indicating improved thermal stability compared to the control. F2 presented a DTGₘₐₓ at 250 °C and 50% mass loss at the same temperature.

In contrast, F3 showed improved thermal stability among the blended films, as indicated by the higher temperature required to reach 50% mass loss (271 °C) and its DTGₘₐₓ peak at 249 °C. Although differences in total mass loss were observed among formulations, F3 also exhibited the highest residual mass at 600 °C (27%), suggesting a more stable char-forming structure. These results may be associated with enhanced intermolecular interactions and improved barrier properties arising from an optimal balance between pectin and *Opuntia ficus-indica* cladode powder at this formulation.

F4 followed a similar trend, with DTGₘₐₓ at 244 °C and 50% weight loss at 265 °C, reinforcing its relatively higher thermal resistance compared to F1 and F2. At 600 °C, residual mass values ranged from 18% (F2) to 27% (F3), indicating that formulation composition significantly influenced thermal degradation behavior and char formation. Such residues reflect the portion of the material that withstood thermal degradation, often attributed to inorganic ash or persistent aromatic structures [25].

In summary, F3 and F4 films demonstrated improved thermal resistance, as reflected by higher decomposition onset temperatures and higher residual mass. All films remained thermally stable up to at least 135–145 °C, that is, the temperature above which the films start to decompose. This thermal stability may be relevant for food packaging applications involving moderate thermal exposure.

### Structural and Morphological Characterization of the Films

**Fig. 2:**
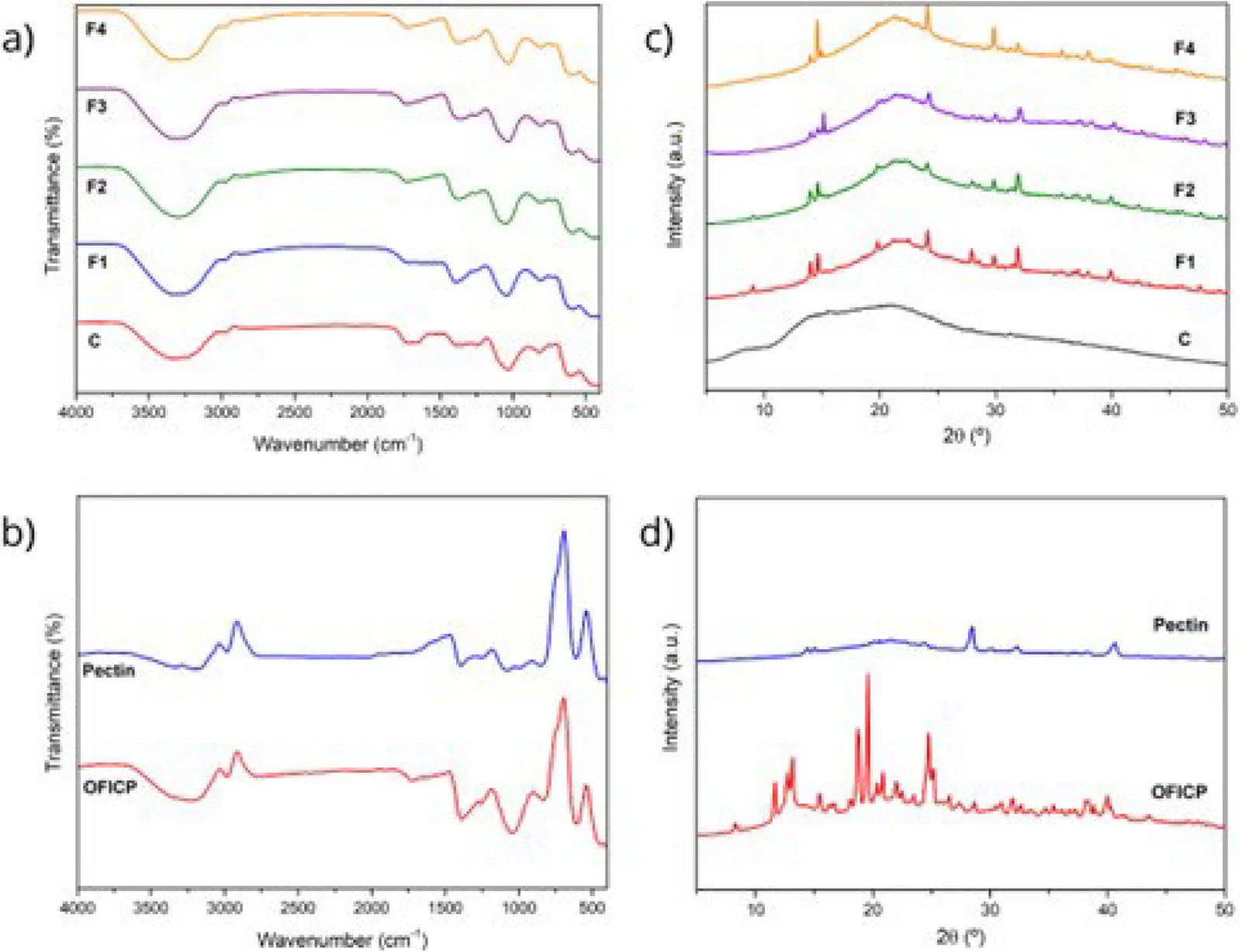
Infrared spectroscopy (FTIR) of the (**a**) films; and (**b**) raw materials; and XRD patterns of (**c**) films; and (**d**) raw materials

FTIR is widely employed to elucidate the chemical structure of biopolymer films, particularly to identify functional groups and evaluate potential crosslinking interactions [4]. The FTIR spectra of the individual components and composite films (Fig. 2a and b) revealed a broad absorption band between 3,200 and 3,600 cm⁻¹, attributed to O–H stretching vibrations. This feature, characteristic of hydroxyl groups, is commonly associated with both OFICP and pectin, reflecting their inherently hydrophilic nature [5].

*C = control film (2 g pectin); F1= (0.5 g pectin + 2 g of OFICP), F2 = (1.0 g pectin + 2 g of OFICP), F3 = (1.5 g pectin + 2 g of OFICP), F4 = (2 g pectin + 2 g of OFICP).

A weaker absorption band near 2920 cm⁻¹ was consistently present in all samples, corresponding to the asymmetric stretching of CH and CH₂ groups [26]. In the control film (C) and pure pectin spectrum, a distinct peak around 1740 cm⁻¹ was observed, which is indicative of C = O stretching vibrations of esterified carboxyl groups. This band is typically used to assess the degree of methoxylation in pectin [5]. In contrast, this peak was much less pronounced in the OFICP spectrum, suggesting a lower degree of esterification or differences in the carboxyl group environment. Further bands between 1600 and 1400 cm⁻¹ were more prominent in the OFICP and blended films, corresponding to asymmetric and symmetric stretching of COO⁻ groups. These are typically related to uronic acids present in cactus mucilage; therefore, in the OFICP sample [6]. Additionally, the spectral region from 1,320 to 1,025 cm⁻¹ is characteristic of C–N, P–OH, and –COOH groups, indicating the presence of aromatic proteins, phosphoric groups, and polysaccharides [4].

Notably, the bands between 900 and 850 cm⁻¹, especially intense in the OFICP spectrum, suggest the presence of α- and β-D-glucose configurations, which further support the sugar configuration of *Opuntia fícus-indica* polyssacharides [5]. The FTIR results confirm the chemical complexity of the formulations and indicate intermolecular interactions between OFICP and pectin. These interactions are likely to impact not only the films’ structural integrity but also their functional attributes, such as barrier performance.

X-ray diffraction (XRD) patterns of OFICP/Pectin films, OFICP, and pectin are shown in Fig. 2c and d. XRD is widely used to assess the structural features and miscibility of polymer blends, contributing to the structural and technological characterization of the films. When components are immiscible or exhibit low compatibility, distinct crystalline domains corresponding to each polymer may be observed [27]. Overall, the OFICP/Pectin films exhibited characteristic peaks associated with both *Opuntia ficus-indica* cladode powder and pectin. The increase in pectin content did not appear to significantly influence the structural characteristics of the blends, as formulations F1 to F4 displayed similar profiles.

The OFICP sample exhibited sharp, intense peaks at 2θ = 15°, 25°, and 30°, indicating a high degree of crystallinity [14]. The peaks at 2θ ≈ 14.9°, 15.2°, and 24.3° can be attributed to cellulose I, while additional reflections are associated with calcium oxalate monohydrate (Ca(C₂O₄)·H₂O), a mineral compound naturally present in *Opuntia ficus-indica* cladode tissue [4]. By contrast, the pectin diffraction pattern was more diffuse, displaying a broad, low-intensity peak around 2θ = 20°, which is characteristic of amorphous materials [4]. Similarly, the OFICP/pectin films displayed a broader, less intense peak at 2θ = 19°, consistent with reduced crystallinity resulting from pectin incorporation. This suggests that blending with pectin disrupts the ordered structure of OFICP, resulting in an amorphous configuration in the films. Thus, there are no new relevant peaks, suggesting mainly physical interaction rather than a new crystalline structure. The control film (C) is more amorphous, and F4 is the most crystalline.

Regarding morphological characterization, Fig. S3 shows the micrographs of the surface and fracture (cross-section) of the control and composite films, as well as their main raw material (OFICP and pectin). SEM provides insights into the relationship between the films’ physicochemical properties and their microstructure [13]. SEM micrographs of both OFICP and pectin showed aggregates and small, irregular particles. Similar results were found by Makhloufi et al [12] and Oudir et al [4] for OFICP micrographs. The surface micrographs showed a rough texture with micron-sized particles and irregularly shaped aggregates. Undulations were also observed on the surfaces of the blend films, as seen in the cross-section. Additionally, all blend films (F1 to F4) exhibited a more irregular and discontinuous cross-sectional morphology compared to the control film. This may be attributed to the fibrous nature of OFICP, as noted by Oudir et al. [4] and de Farias et al. [5].

Furthermore, it has been reported that a higher pectin ratio contributes to a denser film matrix. This is due to stronger interactions within the polymer network as pectin concentration increases, which enhances film compactness and cohesive strength [16]. A more compact film structure is typically associated with increased tensile strength and reduced WVP and elongation at break [14]. Additionally, full proximate composition analysis of OFICP and pectin was not performed in this study and is acknowledged as a limitation, representing an opportunity for future investigation to further elucidate the compositional contributions to the observed structural and mechanical behavior.

### Degradation Assessment

The film’s disintegration behavior was evaluated under both soil and marine conditions. The degradation behavior of the various formulations was qualitatively assessed through visual inspection of the samples over time. Due to the strong adherence of soil particles to the film surfaces, weight loss measurements were not feasible; therefore, visual analysis was adopted as the primary method for monitoring the degradation process.

The films’ structure gradually eroded over time (Fig. S4). By the sixth day of testing, all film samples had undergone significant changes in color and structural integrity, indicating the onset of polymer degradation. This process appears to be mainly driven by environmental humidity and temperature, which cause film swelling and subsequent fragmentation [6] By day 12, films F1 and F2 had almost completely degraded, whereas formulations with a higher pectin content showed greater resistance to decomposition. This phenomenon can be explained by the high affinity of *Opuntia ficus-indica* cladode powder for water: films with a greater hydrophilic character allow moisture and microorganisms to penetrate their structure more easily, consequently accelerating the degradation process [28]. By day 28, all formulations had undergone extensive disintegration, and the remaining film residues were no longer visually distinguishable from the surrounding soil. This disintegration occurred at a substantially faster rate than that typically observed for conventional plastic packaging. These results are consistent with previous findings by Mannai et al [29] and López-Díaz et al [14], who reported similarly rapid breakdown of mucilage-based films made from *Opuntia ficus-indica* and pitaya (*Hylocereus undatus*) within 28–40 days in soil and organic compost, respectively.

In the simulated marine disintegration test, all film samples underwent complete disintegration within 2 h and 55 min. This rapid degradation was anticipated, given the hydrophilic nature of the polymers present in the film matrix, particularly OFICP and pectin.

Although these results provide qualitative evidence of degradation under environmentally relevant conditions, quantitative analyses using standardized methods (e.g., mass loss under controlled composting, CO₂ evolution, or ASTM/ISO biodegradation assays) would be necessary to accurately determine degradation kinetics and enable more comprehensive comparisons with conventional materials.

## Conclusion

This study successfully developed and characterized biodegradable films based on *Opuntia ficus-indica* cladode powder and pectin, emphasizing the relevance of natural macromolecules, particularly polysaccharides, for sustainable packaging applications. The results demonstrate that the combination of OFICP and pectin yields films with adequate mechanical performance, good thermal stability, and effective biodegradability under both soil and marine conditions. Notably, increasing the pectin content enhanced several film properties, with the F3 and F4 formulations achieving the most balanced performance across all evaluations.

Due to their hydrophilic nature and physicochemical characteristics, these films may have potential for use in biodegradable packaging systems and related applications, particularly for products with low moisture content. However, additional studies involving real food models and storage conditions are necessary to confirm their suitability for food packaging applications. Furthermore, the incorporation of underutilized polysaccharides, such as whole Opuntia ficus-indica cladodes, together with commercial pectin, represents a promising strategy for the development of sustainable biomaterials with potential environmental benefits. 

## Supplementary Information

Below is the link to the electronic supplementary material.

Supplementary Material 1

## Data Availability

No datasets were generated or analysed during the current study.
