# Supplementary material for: Characterization of Films Based on Opuntia ficus-indica and Pectin
Source: Plant Foods Hum Nutr. 2026 Sep 23;81(4):122. doi: 10.1007/s11130-026-01570-5 (PMC13601191; doi:10.1007/s11130-026-01570-5)
Supplement: Supplementary file 1 [file 11130_2026_1570_MOESM1_ESM.docx]

**Characterization of films based on *Opuntia ficus-indica* and pectin**

Júlia Matos Coqueiro *^a^*, Júlia Marques Brandão*^b^*, Stefanny Pereira Atanes*^c^*, Sibele Santos Fernandes*^c^*, Deborah Murowaniecki Otero *^ad,*^*

*^a^Graduate Program in Food Science, Faculty of Pharmacy, Federal University of Bahia, Campus Ondina, Salvador, Bahia 40170-290, Brazil.*

*^b^Nutrition School, Federal University of Bahia, Campus Canela, Salvador, Bahia, 40110907, Brazil.*

*^c^Laboratory of Food Technology, School of Chemistry and Food, Federal University of Rio Grande, Rio Grande, RS, Brazil.*

*^d^Graduate Program in Food, Nutrition and Health, Nutrition School, Federal University of Bahia, Campus Canela, Salvador, Bahia, 40110907, Brazil.*

* Contact: Deborah Murowaniecki Otero [deborah.otero@ufba.br](mailto:deborah.otero@ufba.br); deborah.m.otero@gmail.com Graduate Program in Food, Nutrition and Health, Nutrition School, Federal University of Bahia, Campus Canela, Salvador, Bahia, 40110907, Brazil

**Supplementary Material**

**Materials and methods**

**Plant material and reagents**

*Opuntia fícus-indica* cladodes were collected in Boa Nova (Boa Nova, BA, Brazil - 14°23′S 40°03′O). They were washed with tap water, rinsed with distilled water, disinfected with sodium hypochlorite solution at 2% (Sigma Aldrich®, USA), cut into cubes, and frozen in vacuum-sealed packaging until use. Pectin was purchased from Dinamica^®^ Ltda (Indaiatuba, Brazil). Glycerol (99,5%) was purchased from Labsynth (Diadema, Brazil).

**Preparation of *Opuntia ficus-indica* cladodes powder (OFICP)**

The sealed packaging of the *Opuntia ficus-indica* was thawed in a water bath. The cladodes were blended in a 550 W blender (Mondial, Easy Power, Brazil) until a homogeneous mixture formed. The resulting 'juice' was poured into Petri dishes, frozen, and then freeze-dried. The freeze-dried cladodes were sifted through a 0.180 mm mesh sieve until a fine powder was obtained, which was then stored at room temperature in vacuum-sealed packages, protected from light, until further use.

**Film formulation**

The films were prepared through casting, following an adapted procedure from [12]. All formulations contained a fixed amount of *Opuntia ficus-indica* cladode powder (2 g), defined after optimization trials, and glycerol (30% w/w of total solids). Pectin was added at increasing amounts - 0.5 g (F1), 1.0 g (F2), 1.5 g (F3), and 2.0 g (F4). The control formulation (C) consisted solely of 2.0 g of pectin and glycerol (30% w/w), since preliminary tests demonstrated that films prepared solely with OFICP were brittle and non-malleable, preventing further characterization. Each formulation was solubilized in 100 mL of distilled water under constant stirring in an ultra-thermostatic bath (QUIMIS, Q214M2, Brazil) at 90 °C. After adding OFICP, the mixture was stirred for 30 min, followed by the incorporation of glycerol and an additional 15-min stirring period. The film-forming solution (20 g) was then poured into Petri dishes (08 x 08 cm) and dried in an oven with air circulation (QUIMIS, Q316M4, Brazil).

**Film evaluation**

**Moisture content and water activity**

The moisture content of the prepared blend films was measured by a method described by Fernandes et al. [13] Specimens with 1 x 1 cm were weighed (m*i*), and oven-dried (Quimis, model Q314, Brazil) at 105 ± 2 °C for 24 h to obtain the dry matter. The moisture content (%) was determined considering the difference between the dry matter and the initial mass of the film.

The film’s water activity (Aw) was determined in a LabTouch Novasina® water activity analyzer (Novasina, Model LabTouch, Switzerland). Analysis was performed in triplicate, at room temperature.

**Contact angle**

The surface wettability of the prepared films was assessed by the sessile drop method [13]. This method measured the contact angle using a goniometer, which consisted of a CCD camera and image analysis software (ImageJ 1.53k). An electronic pipette deposited a drop of distilled water (10 μL) on the surface of each film. The angle between the drop and the film surface was then recorded immediately upon deposition at ambient room temperature to ensure measurement of the static contact angle. The mean values were calculated in five random droplets for each film.

**Water Vapor Permeability**

The water vapor permeability (WVP) was measured gravimetrically according to the ASTM Standard Method E96-00 [14]. Films were sealed in permeation cells containing silica (desiccant). These cells were stored in desiccators containing distilled water inside, and weight differences were recorded every 24 h for 7 days. To calculate the WVP, the following Equation 2 was used:

$$WVP=\frac{W\times L}{A\times t\times\triangle P}$$

(2)

Where W is the mass gain of the permeation cell (g); L is the sample thickness (mm); A is the exposed area of the film (m^2^); t is the time of mass gain (d); and ΔP is the vapor pressure difference across the sample (kPa).

WVP values were subsequently converted to SI units (g·m⁻¹·s⁻¹·Pa⁻¹) by adjusting thickness (mm to m), time (days to seconds), and pressure (kPa to Pa) accordingly.

**Color parameters**

The films surface color parameters (CIELab scale) were assessed using a colorimeter (Konica Minolta, CR-400, Japan) and determined as L* (lightness: black = 0 and white = 100), a* (green = −a* and red = +a*), and b* (blue = −b* and yellow = +b*). The total color difference of the films (ΔE) was given by the following Equation 3:

$$\triangle E*=\sqrt{\begin{aligned} \left( L*film-L*standard \right)^{2}+\left( a*film-a*standard \right)^{2}+ \\ {(b*film-b*standard)}^{2} \end{aligned}}$$

(3)

Where ΔL*, Δa*, and Δb* are the differences between the color of the white tile and the color of the samples [12]. The reading was taken on three different films for each formulation. The sample's opacity (%) was obtained by calculating the ratio between the black-and-white standards assessed using a colorimeter (Konica Minolta, CR-400, Japan).

**Film thickness**

**T**he thickness (mm) of the films was determined using a digital micrometer (Insize, IP65, Brazil) with a precision of 0.001 mm. Ten measurements were performed at random locations around each sample [6].

**Mechanical properties**

The mechanical properties tensile strength (MPa) and elongation (%) were evaluated according to the ASTM D882-02 methodology [15]. The samples were cut into strips measuring 85 mm in length and 25 mm in width and fixed vertically in grippers coupled to a texturometer (Stable Micro Systems, TA.XTplus, England). The tests were performed in triplicate, for each film formulation, with an initial distance of 50 mm between the grippers and a probe speed of 2 mm.s^-1^.

**Thermal analysis**

Differential scanning colorimetry (DSC), along with thermogravimetric (TGA) analysis, was used to evaluate the thermal characteristics of the samples. To perform DSC (Shimadzu, TA-60WS, Japan), samples were placed in aluminum pans and heated from 10 to 400 °C at a rate of 10 °C.min^-1^. Thermogravimetric analysis (TGA) (Shimadzu, DTG-6, Japan) was performed to verify the weight loss of the films using a thermal analyzer, and aluminum pans were also used, varying the temperature from 20 to 600 °C [16].

**Structural and morphological characterization**

To characterize the morphology of the films, an electron microscope (Jeol, JSM-6610LV, Japan) was used, which provided surface and cross-sectional images by scanning electron microscopy (SEM). For this purpose, the films were fixed to aluminum pins coated with gold at 10 kV [17].

Fourier Transform Infrared Spectroscopy (FTIR) analysis was employed to identify the functional groups of the starting materials and the intermolecular interactions that occurred between them in the blend films. The FTIR spectra of the starting materials and the prepared blend films were recorded using a Fourier transform infrared spectrometer (Spectrum Two FT-IR Spectrometer, USA) in the wavenumber range of 4000–400 cm^−1^ with a resolution of 64 cm^−1^.

The X-ray diffraction (XRD) patterns of the OFICP, pectin, and film samples were obtained by a X-ray diffractometer (PerkinElmer, UATR two, USA), using Cu-Kα radiation (λ = 1.5405 Å), with a voltage of 45 kV and a current of 30 mA. The scanning angle (2θ) range was 5–50º at a scanning rate of 2º / min.

**Disintegration assessment**

The biodegradation test in soil was conducted following the procedure described by López-Díaz et al. [18]. Film samples, cut into 2 × 2 cm squares, were placed in small mesh baskets and buried at a depth of 5 cm in plastic trays filled with regional soil (Lauro de Freitas, BA, Brazil). The trays were maintained under aerobic conditions at room temperature throughout the experiment. Biodegradation was monitored through visual inspection: the samples were retrieved every two days using the basket handles, photographed, and then reburied to continue the evaluation process.

To evaluate biodegradability in seawater, film samples were cut into 1 × 1 cm squares and placed in containers with 50 mL of seawater, which was collected 25 m offshore from Cassino Beach-Rio Grande, RS, Brazil (31°47'02'' to 32°39'45'' south latitude and 52°03'50'' to 52°41'50'' west longitude). The containers were continuously agitated at 150 rpm until the films were completely solubilized [19].

**Statistical analysis**

Statistical analysis was performed to determine the significance of differences between the five film formulations using a one-way analysis of variance (ANOVA) and a post-hoc Tukey’s test with Statistica 5 (Statsoft^@^) software. The significance level was set at p < 0.05.

**Results and Discussion**

**Fig. S1. Visual appearance of the developed films.**

*C = control film (2 g pectin); F1= (0.5 g pectin + 2 g of OFICP), F2 = (1.0 g pectin + 2 g of OFICP), F3 = (1.5 g pectin + 2g of OFICP), F4 = (2 g pectin + 2 g of OFICP).

**Table S1.** Color parameters and opacity of developed films.

| Films | Color parameters |  |  |  | Opacity (%) |
| --- | --- | --- | --- | --- | --- |
|  | L* | a* | b* | ΔE |  |
| C | 90.67 ± 1.38^a^ | 4.59 ± 0.34^a^ | 13.46 ± 2.89^c^ | 17.00 ± 1.92^d^ | 14.94 ± 0.76^b^ |
| F1 | 65.83 ± 2.19^c^ | -2.47 ± 0.26^b^ | 40.63 ± 1.18^a^ | 54.64 ± 1.78^a^ | 25.07 ± 1.96^a^ |
| F2 | 68.97 ± 1.98^bc^ | -2.21 ± 0.03^bc^ | 36.64 ± 1.81^ab^ | 50.73 ± 0.84^ab^ | 24.17 ± 0.85^a^ |
| F3 | 70.92 ± 0.68^b^ | -1.85 ± 0.19^c^ | 31.41 ± 2.64^b^ | 44.20 ± 2.40^c^ | 23.37 ± 0.57^a^ |
| F4 | 69.80 ± 0.55^bc^ | -1.83 ± 0.12^c^ | 37.37 ± 0.64^a^ | 49.48 ± 0.13^b^ | 21.99 ± 2.68^a^ |

L* = brightness; a* and b* = chroma; ΔE = total difference in color; C = control film (2 g pectin); F1= (0.5 g pectin + 2 g of OFICP), F2 = (1.0 g pectin + 2 g of OFICP), F3 = (1.5 g pectin + 2g of OFICP), F4 = (2 g pectin + 2 g of OFICP). Average of three values with standard deviation, the same letter in the column indicates no significant difference between the means by the Tukey test (p < 0.05).

**Fig. S2.** (a) DSC; (b) TGA of formulated films with added OFICP (F1, F2, F3, and F4) and without it (control) and (c) DTG curves of OFICP/pectin films

*C = control film (2 g pectin); F1= (0.5 g pectin + 2 g of OFICP), F2 = (1.0 g pectin + 2 g of OFICP), F3 = (1.5 g pectin + 2g of OFICP), F4 = (2 g pectin + 2 g of OFICP).

**Fig. S3.** Surface and cross-sectional micrographs of OFICP, pectin, and films formulations.

*C = control film (2 g pectin); F1= (0.5 g pectin + 2 g of OFICP), F2 = (1.0 g pectin + 2 g of OFICP), F3 = (1.5 g pectin + 2g of OFICP), F4 = (2 g pectin + 2 g of OFICP).

**Fig. S4.** Biodegradability in soil of control film and incorporated with OFICP and pectin.

*C = control film (2 g pectin); F1= (0.5 g pectin + 2 g of OFICP), F2 = (1.0 g pectin + 2 g of OFICP), F3 = (1.5 g pectin + 2g of OFICP), F4 = (2 g pectin + 2 g of OFICP).

**REFERENCES**

11. Tarique J, Sapuan SM, Khalina A. Effect of glycerol plasticizer loading on the physical, mechanical, thermal and barrier properties of arrowroot (*Maranta arundinacea*) starch biopolymers. *Sci Rep*. 2021;11:13900. <https://doi.org/10.1038/s41598-021-93094-y>

12. Makhloufi N, Chougui N, Rezgui F, Benramdane E, Freire CSR, Vilela C, et al. Bio-based sustainable films from Algerian *Opuntia ficus-indica* cladodes powder: Effect of plasticizer content. *J Appl Polym Sci*. 2021;138(20). <https://doi.org/10.1002/app.50450>

13. Fernandes SS, Romani VP, da Silva Filipini G, Martins VG. Chia seeds to develop new biodegradable polymers for food packaging: Properties and biodegradability. *Polym Eng Sci*. 2020;60(9):2214–2223. <https://doi.org/10.1002/pen.25464>

14. ASTM International. Standard test methods for water vapor transmission of materials (E96-00). West Conshohocken, PA; 2000. <https://doi.org/10.1520/E0096-00>

15. ASTM International. Standard test method for tensile properties of thin plastic sheeting (D882-02). West Conshohocken, PA; 2002. <https://doi.org/10.1520/D0882-02>

16. da Silva Filipini G, Romani VP, Guimarães Martins V. Biodegradable and active-intelligent films based on methylcellulose and jambolão (*Syzygium cumini*) skins extract for food packaging. *Food Hydrocoll*. 2020;109:106139. <https://doi.org/10.1016/j.foodhyd.2020.106139>

17. Santos LG, Alves-Silva GF, Martins VG. Active-intelligent and biodegradable sodium alginate films loaded with *Clitoria ternatea* anthocyanin-rich extract to preserve and monitor food freshness. *Int J Biol Macromol*. 2022;220:866–877. <https://doi.org/10.1016/j.ijbiomac.2022.08.120>

18. López-Díaz AS, Barriada-Bernal LG, Rodríguez-Ramírez J, Méndez-Lagunas LL. Characterization of pitahaya (*Hylocereus undatus*) mucilage-based films. *Appl Food Res*. 2023;3(1):100266. <https://doi.org/10.1016/j.afres.2023.100266>

19. ASTM International. Standard test method for measurement of aqueous solubility (E1148). West Conshohocken, PA; 2002. <https://doi.org/10.1520/E1148-02>

20. Li Y, Duan Q, Yue S, Alee M, Liu H. Enhancing mechanical and water barrier properties of starch film using chia mucilage. *Int J Biol Macromol*. 2024;274:133288. <https://doi.org/10.1016/j.ijbiomac.2024.133288>

21. Tristanto NA, Cao W, Chen N, Suryoprabowo S, Soetaredjo FE, Ismadji S, et al. Pectin extracted from red dragon fruit (*Hylocereus polyrhizus*) peel and its usage in edible film. *Int J Biol Macromol*. 2024;276:133804. <https://doi.org/10.1016/j.ijbiomac.2024.133804>

23. Marques de Farias P, Barros de Vasconcelos L, da Silva Ferreira ME, Alves Filho EG, de Freitas VAA, Tapia-Blácido DR. Nopal cladode as a novel reinforcing and antioxidant agent for starch-based films: A comparison with lignin and propolis extract. *Int J Biol Macromol*. 2021;183:614–626. <https://doi.org/10.1016/j.ijbiomac.2021.04.143>

24. Guo S, Liang D, Liu Q, Luo H, Liang W, Li W. Cinnamaldehyde emulsions for a composite film based on polyvinyl alcohol/hydrophobically modified konjac glucomannan matrix: Degradability and bacteriostatic properties. *Food Packag Shelf Life*. 2024;44:101318. <https://doi.org/10.1016/j.fpsl.2024.101318>

25. Gan L, Jiang G, Yang Y, Zheng B, Zhang S, Li X, et al. Development and characterization of levan/pullulan/chitosan edible films enriched with ε-polylysine for active food packaging. *Food Chem*. 2022;388:132989. <https://doi.org/10.1016/j.foodchem.2022.132989>

26. Romani VP, Prentice-Hernández C, Martins VG. Active and sustainable materials from rice starch, fish protein and oregano essential oil for food packaging. *Ind Crops Prod*. 2017;97:268–274. <https://doi.org/10.1016/j.indcrop.2016.12.026>

27. Koirala P, Thuanthong A, Sablani SS, Nirmal N. Formulation and characterization of green tea spent extract incorporated crude tangerine pectin/gelatin active packaging film. *Ind Crops Prod*. 2025;238:122347. <https://doi.org/10.1016/j.indcrop.2025.122347>

28. Jridi M, Abdelhedi O, Salem A, Kechaou H, Nasri M, Menchari Y. Physicochemical, antioxidant and antibacterial properties of fish gelatin-based edible films enriched with orange peel pectin: Wrapping application. *Food Hydrocoll*. 2020;103:105688. <https://doi.org/10.1016/j.foodhyd.2020.105688>

29. Thomas J, Selvin Thomas P, Stephen R. Improved mechanical, thermal and barrier properties of halloysite nanotubes and nanocellulose incorporated PVA–PEO films for food packaging applications. *Food Packag Shelf Life*. 2024;46:101373. <https://doi.org/10.1016/j.fpsl.2024.101373>

30. Fernandes SS, Greque L, Santos MFC, de Novais LMR, D’Oca CDRM, Prentice C, et al. Effect of the spray drying conditions on the physicochemical and structural characteristics and the stability of chia oil microparticles. *J Appl Polym Sci*. 2021;138(39). <https://doi.org/10.1002/app.51015>

31. Gheribi R, Puchot L, Verge P, Jaoued-Grayaa N, Mezni M, Habibi Y, et al. Development of plasticized edible films from *Opuntia ficus-indica* mucilage: A comparative study of various polyol plasticizers. *Carbohydr Polym*. 2018;190:204–211. <https://doi.org/10.1016/j.carbpol.2018.02.085>

32. Kurt A, Toker OS, Tornuk F. Effect of xanthan and locust bean gum synergistic interaction on characteristics of biodegradable edible film. *Int J Biol Macromol*. 2017;102:1035–1044. <https://doi.org/10.1016/j.ijbiomac.2017.04.081>

33. Sha J, Zhu Z, Lin M, Zhu L, Wang Z, Xiao K. Chitosan/MXene/ε-polylysine-based biodegradable packaging films with synergistic photothermal antimicrobial properties. *Food Packag Shelf Life*. 2025;50:101544. <https://doi.org/10.1016/j.fpsl.2025.101544>

34. Mannai F, Mechi L, Alimi F, Alsukaibi AKD, Belgacem MN, Moussaoui Y. Biodegradable composite films based on mucilage from *Opuntia ficus-indica* (Cactaceae): Microstructural, functional and thermal properties. *Int J Biol Macromol*. 2023;252:126456. <https://doi.org/10.1016/j.ijbiomac.2023.126456>
